# CytoGTA: A cytoscape plugin for identifying discriminative subnetwork markers using a game theoretic approach

**DOI:** 10.1371/journal.pone.0185016

**Published:** 2017-10-02

**Authors:** S. Farahmand, M. H. Foroughmand-Araabi, S. Goliaei, Z. Razaghi-Moghadam

**Affiliations:** 1 Faculty of New Sciences and Technologies, University of Tehran, Tehran, Iran; 2 College of Science and Mathematics, University of Massachusetts Boston, Boston, Massachusetts, United States of America; 3 Department of Mathematical Sciences, Sharif University of Technology, Tehran, Iran; 4 University of Tehran, Tehran, Iran; Friedrich-Alexander-Universitat Erlangen-Nurnberg, GERMANY

## Abstract

In recent years, analyzing genome-wide expression profiles to find genetic markers has received much attention as a challenging field of research aiming at unveiling biological mechanisms behind complex disorders. The identification of reliable and reproducible markers has lately been achieved by integrating genome-scale functional relationships and transcriptome datasets, and a number of algorithms have been developed to support this strategy. In this paper, we present a promising and easily applicable tool to accomplish this goal, namely CytoGTA, which is a Cytoscape plug-in that relies on an optimistic game theoretic approach (GTA) for identifying subnetwork markers. Given transcriptomic data of two phenotype classes and interactome data, this plug-in offers discriminative markers for the two classes. The high performance of CytoGTA would not have been achieved if the strategy of GTA was not implemented in Cytoscape. This plug-in provides a simple-to-use platform, convenient for biological researchers to interactively work with and visualize the structure of subnetwork markers. CytoGTA is one of the few available Cytoscape plug-ins for marker identification, which shows superior performance to existing methods.

## Introduction

It is commonly acknowledged that genetic perturbations in human cells are the main reason of cancer initiation and progression[1]. Using potential cancer biomarkers, as an objective evidence for the diagnosis and monitoring cancer in earlier stages, provides a valuable opportunity for researchers to detect, cure, or at least delay the progression of cancer in human body[2]. Genetic mutations may bring diverse consequences, including conformational alteration in protein structure, loss or serious changes in protein function and also deregulation of gene expression. There have been numerous studies suggesting differentially expressed genes (DEGs) in cancer versus normal samples, as biomarkers [3–5]. However, the efficiency and reproducibility of identified biomarkers rely extensively on sample size, data quality and heterogeneity of experimental platforms utilized for analysis[6].

To enhance the efficiency and reproducibility of identified biomarkers, more recent methods have leveraged the assumption that “genes associated to a specific disease, tend to have physical interactions among their products or situate in a particular pathway”[7, 8]. According to this hypothesis, a number of methods confined their analysis to the pathway genes and reported dysregulated pathways with high representation of the DEGs. For example, in an approach proposed by Gue et al. [9] each potentially dysregulated pathway was quantified by the mean and the median of the expression levels of its participated genes. In another study, Tomfohr et al. [10] calculated the activity score of a given pathway by the singular value decomposition technique. Furthermore, Su et al. [11] used a log-likelihood approach to identify potential pathway-based markers.

Regarding the reliability and the accuracy of identified biomarkers, the pathway-based methods mainly overtake the ones based on single genes. However, the main deficiency of pathway-based methods is that, a limited number of genes and significant DEGs may be covered by known pathways. To address this shortcoming, several methods have utilized valuable information embedded in protein-protein interaction networks (PPINs) and integrated gene expression profiles with them[12–18]. The first method, aimed at identifying network-based markers using a simulated annealing algorithm, was proposed by Ideker et al. [16]. Network-based markers which are also referred to as subnetwork markers are a set of mainly significant DEGs with high interconnectivity in a PPIN. The identification of subnetwork markers, with remarkable efficiency and reproducibility, has recently attracted much attention and many studies have been motivated to improve the markers [12–18].

Recently, Farahmand et al. [13] proposed a game theoretic approach (GTA) with the objective of extracting reliable subnetwork markers with potential prognostic utility. The identified subnetwork markers by GTA showed significantly high discriminative power and the proposed strategy of GTA would be applicable to acquire makers linked to a given phenotype. In this paper, a new plugin for Cytoscape [19] (CytoGTA) is presented which uses all GTA features and strengthen to identify optimized subnetwork markers. Cytoscape is an open source tool for integrating, analyzing and visualizing data in the context of networks. CytoGTA plugin takes a PPIN and a gene expression profile of two phenotype classes as input, and returns subnetwork markers that can discriminate two classes. The plugin has been implemented in Cytoscape application, a known graphical environment that is convenient for researchers in systems biology. The outputs of this plugin can be used by other analysis tools within Cytoscape, for further investigations.

## Materials and methods

As we aimed to provide an easy-to-use tool for identifying potential subnetwork markers, CytoGTA has been developed as a Cytoscape app, written in Java programming language. CytoGTA is based on GTA algorithm which has been approved for its superior reproducibility and reliability as compared with some well-known algorithms. CytoGTA takes as input a weighted (or possibly unweighted) PPIN and a gene expression profile, and results in numbers of connected subnetworks, which are highly associated with the given expression profile.

The game theoretic approach used in GTA, models the task of extracting subnetwork markers as a static game. In the modeled game, nodes of the PPIN are considered as players trying to optimize their utility by choosing the strategy of joining or leaving subnetworks, which are respectively represented by 1 and 0 in the subnetwork state vector. The utility of each player is quantified by a payoff function, which is a combination of a gain function and a loss function. The Nash equilibrium is then calculated and the result state vector represents optimized subnetwork marker. In the following, a brief description of the gain and loss functions is given.

### Gain function

Given any subnetwork *G*_*s*_(*V*_*s*_, *E*_*s*_), there are |*V*_*s*_| = *n* proteins corresponding to *n* genes (Genes = {*g*_1_, *g*_2_, …, *g*_n_}). Also, an expression profile for *m* different samples of two different phenotypes is available. The expression vector $x_{i}=\left(x_{i}^{1},x_{i}^{2},\ldots ,x_{i}^{m}\right)$ contains expression values of gene *g*_*i*_(*i* = 1, 2, …, *n*), where $x_{i}^{j}$ is the expression level of gene *g*_*i*_ in sample *j*. In order to relate the sample phenotypes to their given expression levels, the GTA algorithm computes the log-likelihood ratio (LLR) of each gene to obtain a standard feature vector for that gene. This data is then used in association with phenotype data to determine whether statistically significant correlation can be identified between the expression levels for each gene and the given disease [11]. The LLR of gene *g*_*i*_ for *j*^th^ sample, due to two different phenotype classes is defined by:

LLRi(xij)=log[fi1(xij)fi2(xij)] (1)

where $f_{i}^{1}\left(x_{i}^{j}\right)$ and $f_{i}^{2}\left(x_{i}^{j}\right)$ are the conditional probability density function (PDF) of the expression level of gene *g*_*i*_ under phenotype 1 and phenotype 2, respectively. The *density* function in Apache Commons Math [20] is used to calculate the PDF of the expression levels under different distributions of two phenotypes. Commons Math is a self-contained Java library provided by Apache Foundation, which contains components for mathematical and statistical routines. The vector $LLR_{i}=\left(LLR_{i}\left(x_{i}^{1}\right),LLR_{i}\left(x_{i}^{2}\right),\ldots ,\;LLR_{i}\left(x_{i}^{m}\right)\right)$ for gene *g*_*i*_ contains log-likelihood values of gene *g*_*i*_ for *m* different samples.

A local scoring (LS) function is also defined for each gene *g*_*i*_. By this scoring, GTA tries to find the role of each protein in the subnetwork in connecting DEGs. The LS function for gene *g*_*i*_ with joining strategy is defined as Eq (2),

$$LS_{i}=\sum_{l=1}^{k}t-score(LLR_{i_{l}})$$ (2)

where $g_{i_{1}},g_{i_{2}},\ldots ,\;g_{i_{k}}$ are *k* neighbors of gene *g*_*i*_ in *G*_*s*_, and t-scrore is *t*-test statistic score (independent test) for LLR values. Libraries provided by Apache Commons Math is exploited to perform *t*-test statistical score for two unpaired sample data sets assuming unequal variances. Since a gene with leaving strategy has no neighbor in the subnetwork, its LS value is set to be zero.

Furthermore, to score the connectivity of each subnetwork, a density value is assigned. Suppose *G*_*join*_ = (*V*_*join*_, *E*_*join*_) is an induced graph of *G*_*s*_ on nodes (players) with joining strategy (*V*_*join*_), then the density value is defined by:

$$DE\left(G_{join}\right)=\frac{\sum_{e\in E_{join}}w\left(e\right)}{\left(\begin{array}{c} \left|V_{join}\right|\\ 2 \end{array}\right)}$$ (3)

where *w*(*e*) is the weight of edge *e* (for unweighted PPINs, *w*(*e*) is set to be 1 for all edges in *E*_*join*_). The value of *DE*(*G*_*join*_) is assigned to all genes in *G*_*s*_, irrespective of their chosen strategy.

Finally, the gain function (GF) is determined as Eq (4) for gene *g*_*i*_ in *G*_*s*_, in which α, β and γ are constants:

$$GF\left(i,G_{s},\;G_{join}\right)=\alpha \cdot t-score\left(LLR_{i}\right)+\beta \cdot LS_{i}+\gamma \cdot DE\left(G_{join}\right)$$ (4)

In above equation, *α*, *β* and *γ* are weighting parameters to imply each function's importance. *t* − *score*(*LLR*_*i*_) is the t-test statistics score of the *LLR*_*i*_, which is considered in Eq (4) for gene *g*_*i*_ with any chosen strategy.

### Loss function

The loss function (LF) for gene *g*_*i*_ with joining or leaving strategy is defined in Eq (5), where *δ* is a constant.

$$LF\left(i,G_{s},\;G_{join}\right)=\delta \cdot \left(\left|V_{join}\right|-1\right)$$ (5)

### Payoff function

Eventually, the payoff function (PF) for a given agent *g*_*i*_ and the subnetwork *G*_*s*_ is calculated as follows:

$$PF\left(i,G_{s},\;G_{join}\right)=GF\left(i,G_{s}\right)-LF\left(i,G_{s}\right)$$ (6)

By using a numerical method, GTA algorithm examined different values for constants in payoff function, and the most powerful discriminatory markers were achieved by setting *α* = 1.24, *β* = 1, *γ* = 1 and *δ* = 2. To keep the consistency with GTA, in our developed plugin, the same values are assigned to the constants.

### Main algorithm

In the developed algorithm, proteins (gene products) within the given PPIN are sorted in decreasing order, regarding their *t* − *score*(*LLR*) values. Starting from the highest ranked protein, each of them is chosen as a seed, excluding the ones having degree less than the average degree of the PPIN. Once a seed is chosen, all proteins at distance at most two from the seed, are identified in the PPIN. The starting seed and its close nodes (at most two steps away from it) are considered as the players of the algorithm and form a candidate subnetwork. In the candidate subnetwork, all one-step neighbors of the seed, are selected and sorted in increasing order, regarding their degrees, and are then iteratively removed from the subnetwork while their removal increases the local clustering coefficient of the seed. The local clustering coefficient of a node is defined by the proportion of edges between its first neighbors divided by the number of all possible edges between them. Subsequently, the same exact procedure is applied on the two-step neighbors, the ones which are connected to the remaining one-step neighbors from the previous step.

A Nash equilibrium in a game describes a state with a set of strategies for players in which no player gain a better payoff by changing its own strategy alone[14]. In our case, Nash equilibrium occurs when all nodes play their best responses to the strategies of others. In other words, in this Nash equilibrium, no node can get a better payoff by changing its chosen strategy (change from joining to leaving or vice versa), unilaterally. For a given subnetwork of size |*V*_*s*_| and with two available strategies for each of its nodes, there are $2^{\left|V_{s}\right|}$ possible states to be evaluated. Subsequently, for each state, payoffs for every node are calculated, and eventually an equilibrium state is chosen for the subnetwork. In the case of multiple Nash equilibria, the subnetwork with the highest average absolute *t* − *score*(*LLR*) of its genes, is reported as the result.

**Fig 1:**
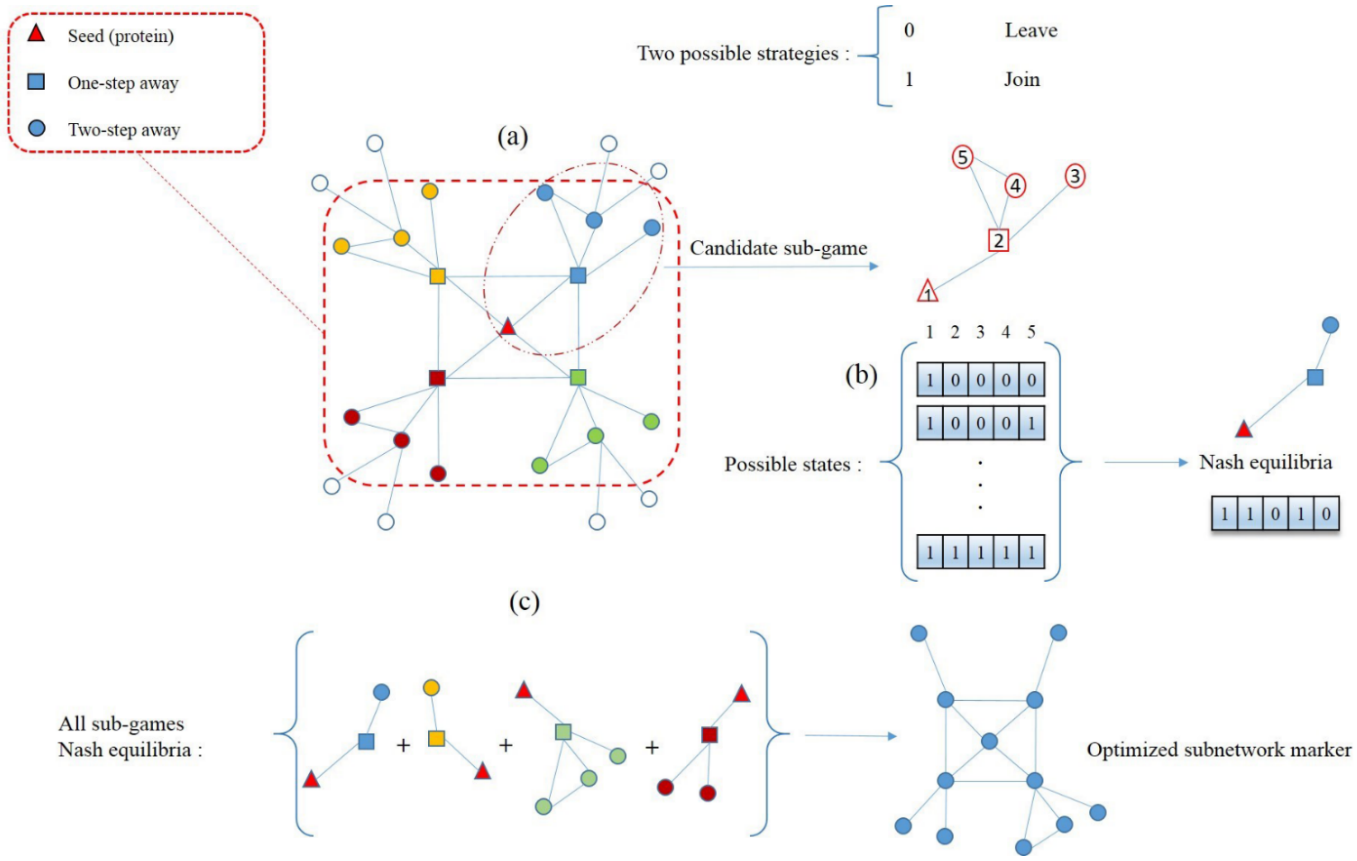
Visual overview of the approach used in GTA algorithm. A candidate subnetwork (game) is shown in (a). The selected seed of this subnetwork (game) has 4 one-step and 12 two-step neighbours. Therefore, the number of possible states to be evaluated, is 2^16^. To reduce the complexity of the game, it is divided into 4 sub-games due to the number of the first-step neighbours (one of these sub-games is surrounded by a dotted line as a representative). Here in this example, each sub-game has 2^4^ possible states. The possible states of the representative sub-game are shown in (b). Since, the seed inevitably takes part in the resulting subnetwork, the value of 1 is assigned to the seed in each possible state. The Nash equilibria of all sub-games are shown in (c) and are then integrated into a single optimized subnetwork marker.

To overwhelm computational limitations, caused by the number of possible states, each game (candidate subnetwork) is divided into a number of sub-games, where each sub-game has a relatively low number of players (nodes). The Nash equilibrium is computed for each sub-game, and then merged, to form a single optimized subnetwork. Although the identified subnetwork may not represent a global Nash equilibrium of the game, it can be considered as a suitable alternative to it. The overview of the game theoretic approach used in GTA is depicted in Fig 1.

To develop CytoGTA, GTA algorithm has been written in Java programming language. Computing the local clustering coefficient and determining the equilibrium for all possible states are two of the most time-consuming parts in CytoGTA. CytoGTA leverages some pervasive characteristics of biological networks, such as small-world property and sparsity, to speed up the computation time in GTA and improve its efficiency. As explained before, in GTA algorithm, for a particular candidate subnetwork, the local clustering coefficient of its seed needs to be updated when any of its one- or two-step neighbors is removed from the subnetwork. In CytoGTA, the algorithm is adapted so that updating the local clustering coefficient is performed more efficiently. Moreover, some minor optimization changes are made to accelerate computing the equilibrium, which leads to the slightly better performance of CytoGTA.

## Results and discussion

**Fig 2:**
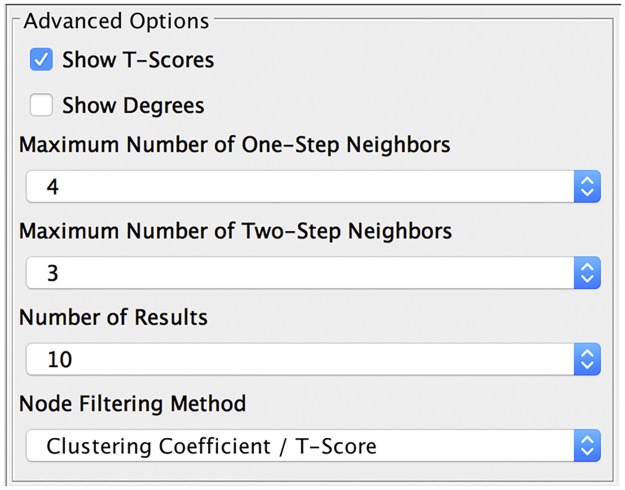
Advanced options in the control panel of CytoGTA. Attributes which can be specified by the user.

To run CytoGTA, two types of data need to be imported into Cytoscape, a PPIN and an expression profile. Using the “Import Network From File” option, the input PPIN (weighted or unweighted network) can be loaded and build in Cytoscape. The expression profile of two phenotype classes is also importable through the “Import Table From File” option. Once the input PPIN and the expression profile are successfully loaded, a summary will appear in “Table Panel”. Moreover, by choosing “CytoGTA” in “Control Panel”, the loaded network is accessible through the “Reference Network” icon, and the list of sample labels in the expression profile are presented in the “Control” and the “Case” icons. To get the mappings between the expression profile of each gene and its corresponding protein in the PPIN, the same system of nomenclature needs to be used in the expression profile and in the PPIN. A number of options are provided in “Advanced Options”, which are expected to be specified before running CytoGTA. Fig 2 shows all CytoGTA icons in “Control Panel”.

To run CytoGTA, it is necessary to select a PPIN from the drop-down list in the “Reference Network” icon, and some samples of different phenotype classes from “Control” and “Case” to be compared by their expression patterns. The further step is to specify the options in “Advanced Options”, which are already initialized with default values. Selecting the checkboxes next to “Show T-Scores” and “Show Degrees” enables CytoGTA to present the t-score and the degree for each gene in the result subnetwork marker. As it is mentioned before, the one-step and the two-step neighbors of the selected seed are pruned iteratively, with the aim of increasing its local clustering coefficient. Furthermore, CytoGTA allows the users to set the maximum values for the numbers of one-step and two-step neighbors, which can vary from 1 to 19. Moreover, the number of result subnetwork markers is selectable through the drop-down list in the "Number of Results". Finally, by “Node Filtering Method” icon, users are provided with the option to select the pruning strategy used by CytoGTA to exclude less informative nodes.

**Fig 3:**
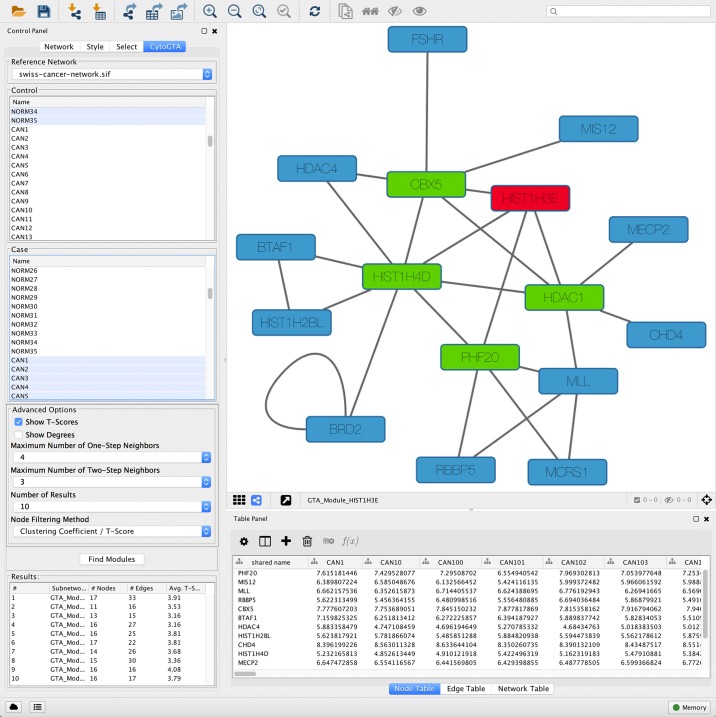
A sample of CytoGTA result. In this sample the maximum numbers of one-step and two-step neighbors are set to be 4 and 3, respectively.

By clicking on “Find Modules”, CytoGTA starts running the algorithm on the loaded input, considering user-requested options. When the execution of CytoGTA is finished, a list of top optimized subnetwork markers appears in the “Results” section of the “Control Panel”. The result list contains the desired number of subnetworks, requested by the user. Each subnetwork marker has given a name, which is descriptive of its seed gene. CytoGTA provides the users with the visualization of the result subnetwork markers, as well as displaying the t-score and the degree of their comprising genes (only if their corresponding checkboxes are ticked). In the graphical visualization of the subnetworks, the seed, the one-step neighbors and the two-step neighbors are distinguished by different colors to enhance the interpretation of the results. The result subnetworks are exportable, and are usable by other analysis tools within Cytoscape. Fig 3 shows different panels obtained from the execution of CytoGTA on a sample data, by setting the maximum numbers of one-step and two-step neighbors to be 4 and 3, respectively.

### Experiments

To evaluate the efficiency of CytoGTA, it was applied on three independent gene expression profiles of metastatic and non-metastatic breast cancer samples, and subsequently, the classification performance of result markers was evaluated by the support vector machine (SVM) method. Two of the expression profiles were retrieved from the Affymetrix U133A platform and are accessible through the Gene Expression Omnibus (GEO) database with GEO accession number GSE7390 [21] (the Belgium dataset) and GSE1456 [22] (the Sweden dataset). The third dataset (the Netherland dataset) was profiled on the Agilent microarray platform, and obtained from the study by van de Vijver et al. [4]. To account for the magnitude differences among transcript levels, all expression profiles were normalized before importing into Cytoscape. The normalization process was performed with the RMA algorithm [23] using the Affy package in R. Furthermore, the human PPIN used in this evaluation, was taken from the study by Lage et al.[24], which contains169.810 high confidence interactions on 12879 proteins.

Approaches used in this study for evaluating the efficiency of CytoGTA, can be categorized into single gene-based, pathway-based and network-based (the greedy and the optimally discriminative (OptDis) methods). For the single gene-bases approach, the 50 genes with the best absolute *t* − *score*(*LLR*) values were chosen as the markers. In the pathway-based approach, among 1320 reported pathways in the C2 curated gene sets in the Molecular Signature Database (MsigDB), the top 50 were extracted based on the *t* − *score*(*LLR*) values of their member genes. The top 50 optimized subnetworks were chosen in network-based approaches employed in this study (greedy, OptDis and CytoGTA). All aforementioned marker types were used to train and test the SVM classifier.

**Fig 4:**
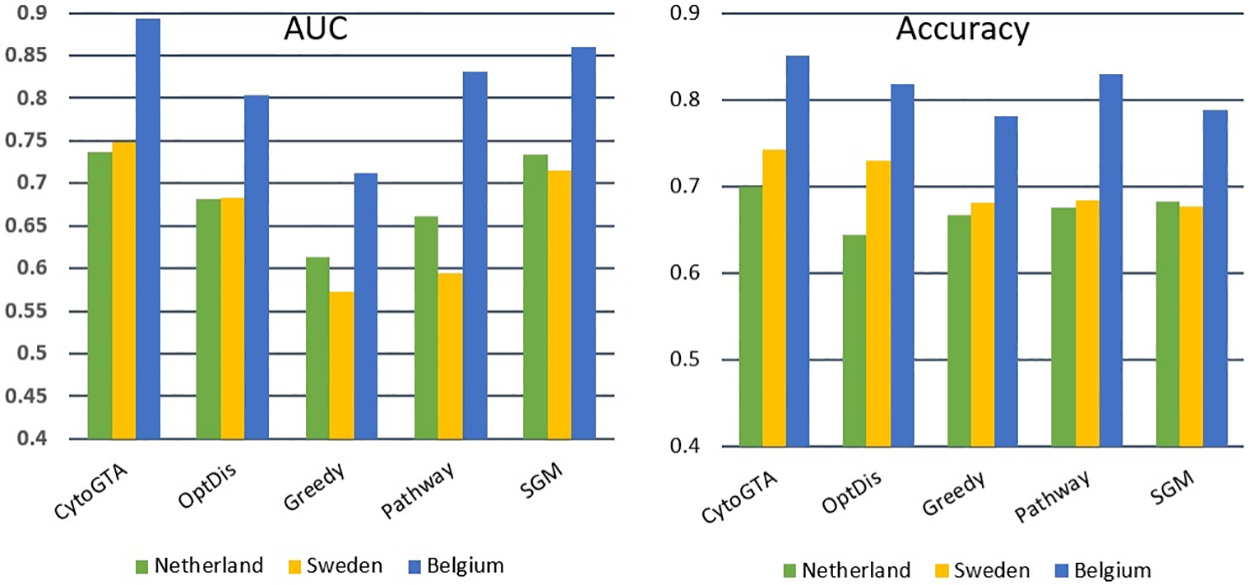
Classification performance comparison of the within-dataset experiment.

Both within-dataset and cross-dataset analyses were conducted to evaluate the classification performance of the markers. The within-dataset classification evaluation was performed by five-fold cross-validation, repeated ten times. The classification performance was measured in terms of accuracy and the area under the curve (AUC). As it is shown in Fig 4, based on both criteria, CytoGTA shows better classification performance compared to other methods.

**Fig 5:**
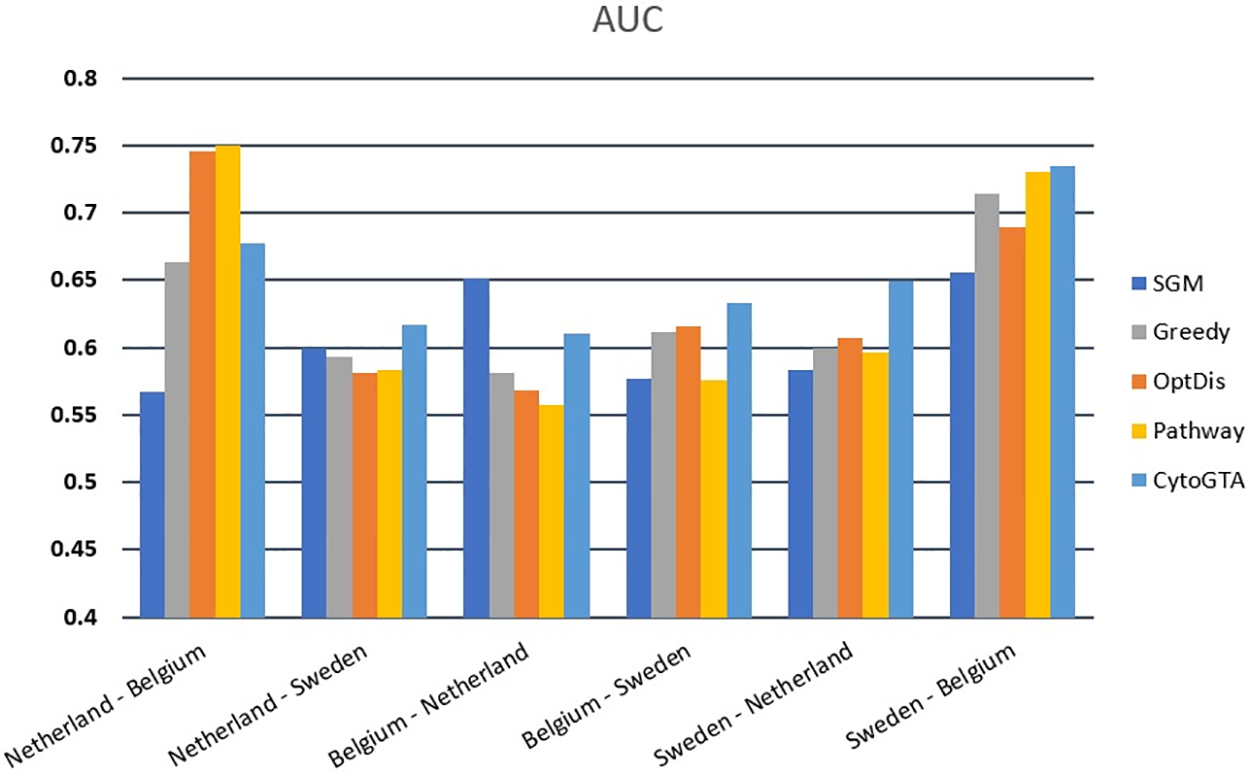
Classification performance comparison of the cross-dataset experiment in term of AUC criterion.

**Fig 6:**
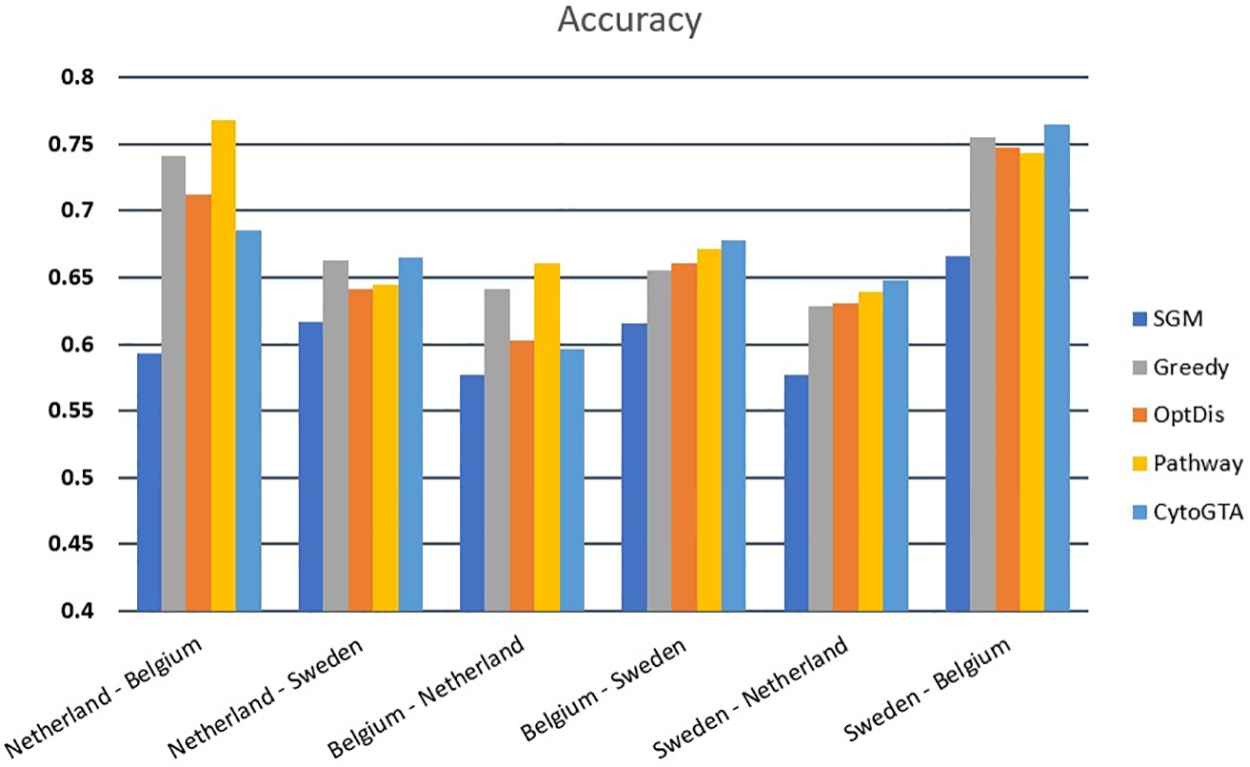
Classification performance comparison of the cross-dataset experiment in term of accuracy criterion.

In addition, the reproducibility of the markers was estimated by the cross-dataset analysis. To this aim, the top 50 markers extracted from one dataset, were used in five-fold cross-validation experiment on the other two datasets. In this analysis, the validation experiment was also repeated ten times. As it is presented in Figs 5 and 6, the classifier constructed on CytoGTA, mostly outperformed those based on other methods, which suggests the superior reproducibility of markers obtained by CytoGTA.

**Fig 7:**
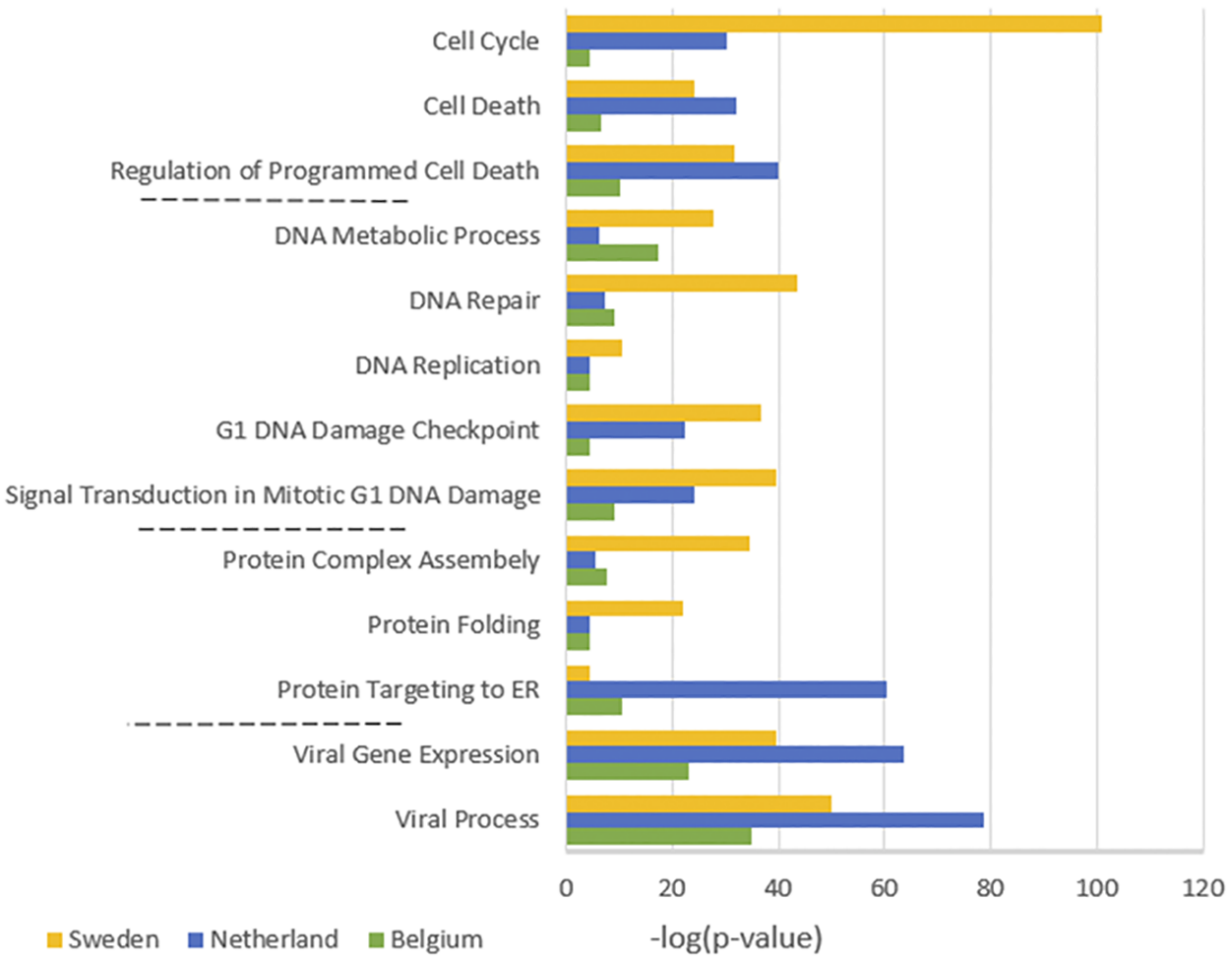
Enriched biological processes of the identified subnetwork markers for Sweden, Netherland and Belgium datasets.

Furthermore, to examine the biological relevance of identified markers with cancer, Gene Ontology (GO) analysis was performed using PANTHER[25]. Interestingly, the result markers were significantly enriched with GO terms (see Fig 7), most of which are well-known in cancer pathology and are consistent with several studies on this field [12, 21, 26].

## Conclusion

In in-silico systems biology, there has been an increasing interest in developing tools, with the objective of facilitating the study of complex diseases and traits. Here, CytoGTA as a new Cytoscape plugin is introduced, in which a PPIN and a gene expression profile of two phenotype classes are given as input, and a number of discriminative markers are returned as output. The high performance of CytoGTA, in terms of reproducibility and reliability, makes it extremely convenient for the purpose of identifying biomarkers in different phenotypes. In this light, embedding CytoGTA in Cytoscape will contribute with the overall profitability of Cytoscape as a comprehensive tool for the analysis of biological networks.

## Availability

The tutorial, full package and related examples are available at https://github.com/cocoamilk/GTA.
